# *vhcub*: Virus-host codon usage co-adaptation analysis

**DOI:** 10.12688/f1000research.21763.1

**Published:** 2019-12-23

**Authors:** Ali Mostafa Anwar, Mohamed Soudy, Radwa Mohamed

**Affiliations:** 1 Department of Genetics, Faculty of Agriculture, Cairo University, Cairo, 12613, Egypt; 2 Bioinformatics program, Faculty of Computer Science, Ain Shams University, Ain Shams, Egypt

**Keywords:** Evolution, Natural selection, Adaptation, Viruses, Codon Usage Bias, R, RStudio

## Abstract

Viruses show noticeable evolution to adapt and reproduce within their hosts. Theoretically, patterns and factors that affect the codon usage of viruses should reflect evolutionary changes that allow them to optimize their codon usage to their hosts. Some software tools can analyze the codon usage of organisms; however, their performance has room for improvement, as these tools do not focus on examining the codon usage co-adaptation between viruses and their hosts. This paper describes the *vhcub *R package, which is a crucial tool used to analyze the co-adaptation of codon usage between a virus and its host, with several implementations of indices and plots. The tool is available from: https://cran.r-project.org/web/packages/vhcub/.

## Introduction

During the translation process from mRNAs to proteins, information is transmitted in the form of triple nucleotides, named codons, which encode amino acids. Multiple codons that encode one amino acid are known as synonymous codons. Studies concerning different organisms report that synonymous codons are not used uniformly within and between genes of one genome, a phenomenon known as codon usage bias (CUB) ^1, 2^. Since viruses rely on the tRNA pool of their hosts in the translation process, previous studies suggest that translation selection or/and directional mutational pressure act on the codon usage of the viral genome to optimize or deoptimize it towards the codon usage of their hosts ^3, 4^.

Tools and packages are available to analyze codon usage, e.g. coRdon ^5^, but there is no package available that focuses on the examination of codon usage co-adaptation between viruses and their hosts. vhcub is a package implemented in R, which aims to easily analyze the co-adaptation of codon usage between a virus and its host. vhcub measures several codon usage bias measurements, such as effective number of codons (ENc) ^6^, codon adaptation index (CAI) ^7^, relative codon deoptimization index (RCDI) ^8^, similarity index (SiD) ^9^, synonymous codon usage orderliness (SCUO) ^10^, and relative synonymous codon usage (RSCU) ^10^. It also provides a statistical dinucleotide over- and under-representation with three different models.

## Methods

### Implementation

vhcub imports Biostrings ^11^, seqinr ^12^ and stringr ^13^ to handle fasta files and manipulate DNA sequences. Also, it imports coRdon ^5^, which is used to estimate different CUB measures.

vhcub first converts the fasta format to data.frame type, to efficiently maintain and calculate different indices implemented in the package. Table 1 describes all the functions available in vhcub, and the result returned from each. Also, it contains references to the equations used to estimate each measure. Furthermore, vhcub uses ggplot2 ^14^ to visualize two important plots named ENc-GC3 plot ( Figure 2) and PR2-plot ( Figure 3), which help to explain the factors influencing a virus’s evolution concerning its CUB.

**Table 1.** Functions available in vhcub, and the result returned from each one.

Function name	Description	Value
fasta.read	Read fasta formate and convert it to data frame	A list with two data.frames; the first one for virus DNA sequences and the second one for the host.
CAI.values	Measure the Codon Adaptation Index (CAI) using Sharp and Li (1987) ^7^ equation, of DNA sequence.	A data.frame containing the computed CAI values for each DNA sequences within df.fasta.
dinuc.base	A measure of statistical dinucleotide over- and under-representation; by allows for random sequence generation by shuffling (with/without replacement) of all bases in the sequence ^13^.	A data.frame containing the computed statistic for each dinucleotide in all DNA sequences within df.virus.
dinuc. codon	A measure of statistical dinucleotide over- and underrepresentation; by allows for random sequence generation by shuffling (with/without replacement) of codons ^13^.	A data.frame containing the computed statistic for each dinucleotide in all DNA sequences within df.virus.
dinuc. syncodon	A measure of statistical dinucleotide over- and underrepresentation; by allows for random sequence generation by shuffling (with/without replacement) of synonymous codons ^13^.	A data.frame containing the computed statistic for each dinucleotide in all DNA sequences within df.virus.
ENc.values	Measure the Effective Number of Codons (ENc) of DNA sequence. Using its modified version (Novembre, 2002) ^6^.	A data.frame containing the computed ENc values for each DNA sequences within df.fasta.
GC.content	Calculates overall GC content as well as GC at first, second, and third codon positions.	A data.frame with overall GC content as well as GC at first, second, and third codon positions of all DNA sequence from df.virus.
RCDI.values	Measure the Relative Codon Deoptimization Index (RCDI) ^8^ of DNA sequence.	A data.frame containing the computed ENc values for each DNA sequences within df.fasta.
RSCU. values	Measure the Relative Synonymous Codon Usage (RSCU) ^7^ of DNA sequence.	A data.frame containing the computed RSCU values for each codon for each DNA sequences within df.fasta.
SCUO. values	Measure the Synonymous Codon Usage Eorderliness (SCUO) of DNA sequence using Wan *et al.*, 2004 ^10^ equation.	A data.frame containing the computed SCUO values for each DNA sequences within df.fasta.
SiD.value	Measure the Similarity Index (SiD) between a virus and its host codon usage ^15^.	A numeric represent a SiD value.
PR2.plot	Make a Parity rule 2 (PR2) plot ^16^, where the AT-bias [A3/(A3 +T3)] at the third codon position of the four- codon amino acids of entire genes are the ordinate and the GC-bias [G3/(G3 +C3)] is the abscissa. The centre of the plot, where both coordinates are 0.5, is where A = U and G = C (PR2), with no bias between the influence of the mutation and selection rates.	A ggplot object.
ENc. GC3plot	Make an ENc-GC3 scatterplot ^17^. Where the y-axis represents the ENc values and the x-axis represents the GC3 content. The red fitting line shows the expected ENc values when codon usage bias affected solely by GC3.	A ggplot object.

### Operation

vhcub was developed using R and is available on CRAN. It is compatible with Windows, and major Linux operating systems. The package can be installed as:


install.packages( "vhcub" )


**Figure 1.**
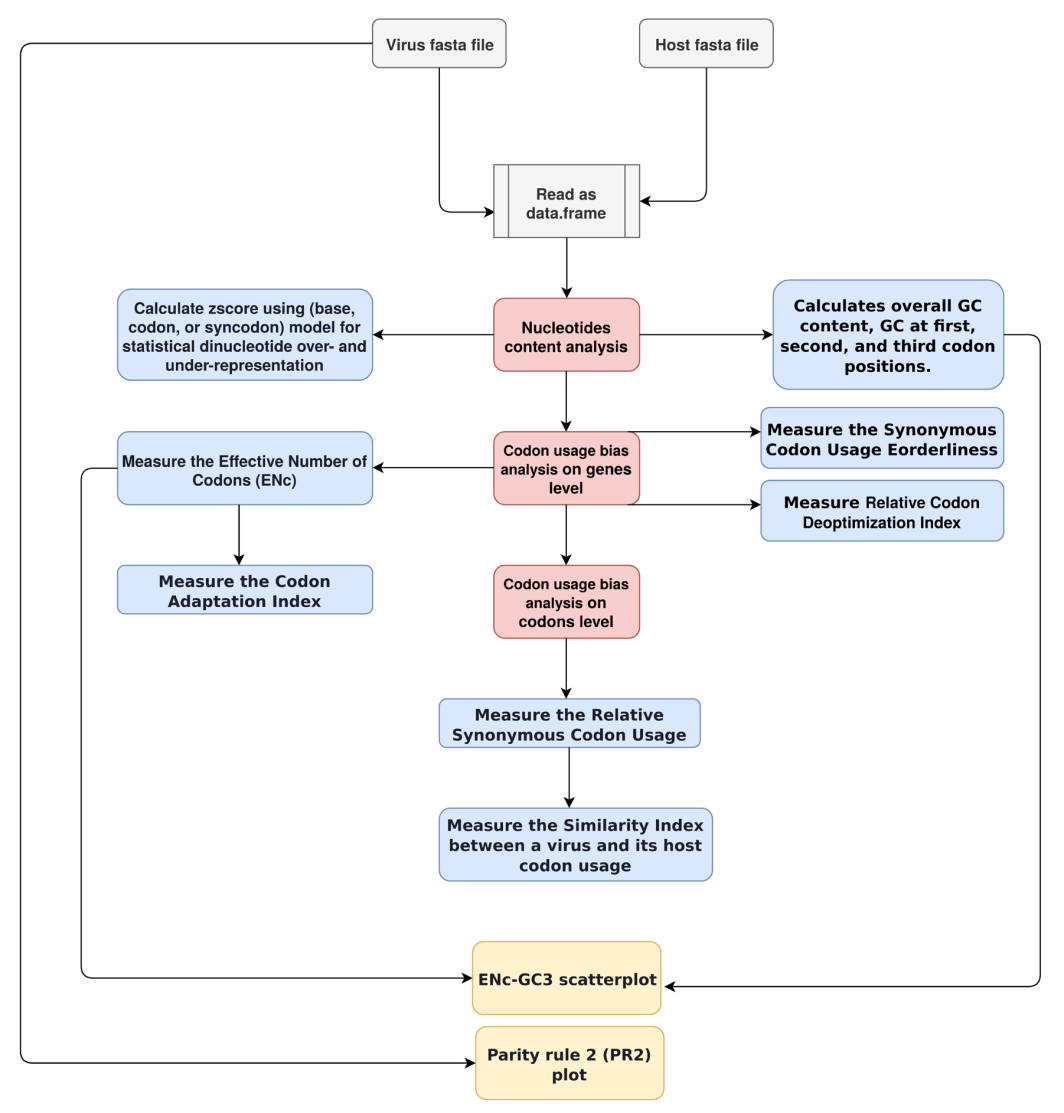
vhcub workflow, to analyze virus-host codon usage co-adaptation. The white boxes represent the input fasta files. The red boxes represent three main analysis, each with different measures (the blue boxes), and the orange boxes represent ENc-GC3 plot and PR2-plot.

Figure 1 describes the vhcub workflow. It starts with reading the fasta files for a virus and its host. After, nucleotide content analysis, codon usage bias analysis on genes and codon level (marked by the red boxes in Figure 1) can be applied independently (the blue boxes in Figure 1). However, within the same analysis, some measures rely on others. For example, the reference set of genes used to estimate a virus codon adaptation index was defined based on the effective number of codons of its host. Finally, the orange boxes in Figure 1 represent the two plots (ENc-GC3 plot and PR2-plot).

**Figure 2.**
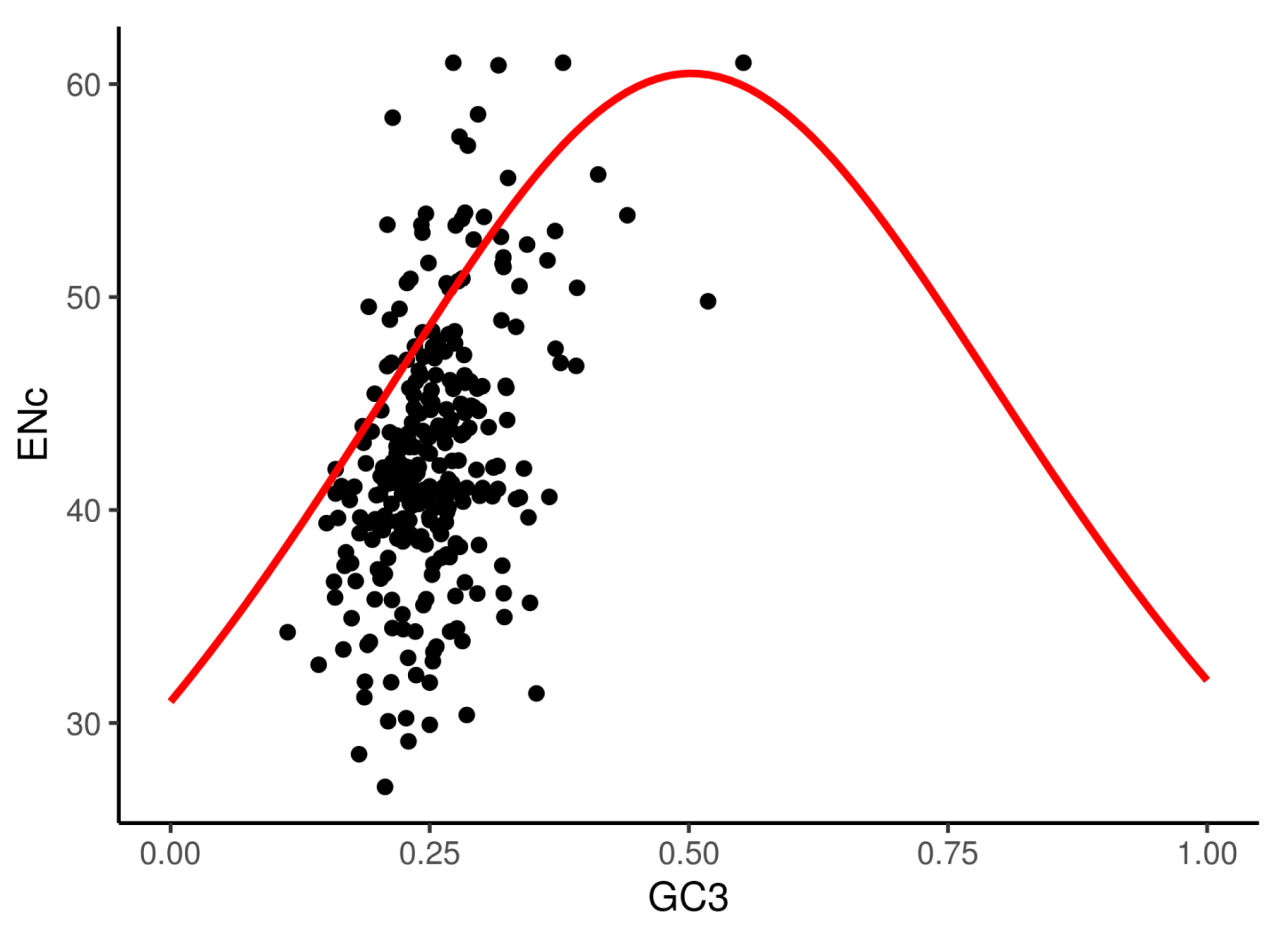
ENc-GC3 plot showing the values of the ENc versus the GC3 content for the virus (Escherichia virus T4) CDS, the solid red line represents the expected ENc values if the codon bias is affected by GC3s only.

**Figure 3.**
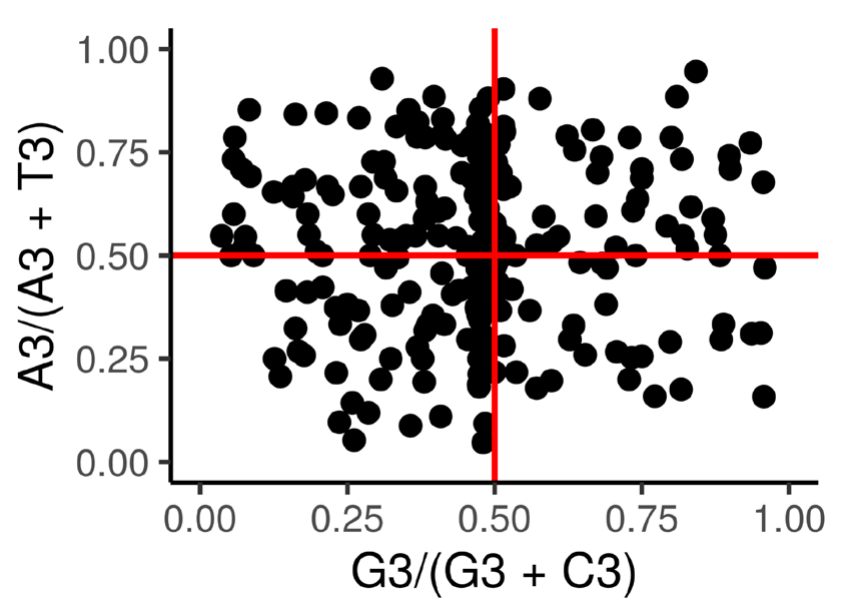
PR2-plot showing CDS of the virus (Escherichia virus T4), plotted based on their GC bias [G3/(G3 + C3)] and AT bias [A3/(A3 + T3)] in the third codon position, the two solid red lines represent both coordinates (ordinate and abscissa) equal to 0.5, where A = T and G = C.

## Use cases

Using vhcub to study the CUB of a virus, its host and the co-adaptation between them is straightforward. As an example, we have used the coding sequences for *Escherichia virus T4* and its host *Escherichia coli* in the form of fasta format.


# First to call the library
library("vhcub")

# To read both files at the same time as a data.frame
# Using fasta.read() function
# virus.fasta = directory path to the virus fasta file
# host.fasta = directory path to the host fasta file.

fasta <− fasta.
                    read(virus.fasta = "EscherichiavirusT4.fasta",
                     host.fasta = "Escherichiacoli.fasta")

fasta.T4 <− fasta[[1]]
fasta.Ecoli <− fasta[[2]]



As mentioned before, each category of analysis could be applied independently. Hence, this example will show only the codon usage bias analysis at the codon level.


# To estimate the similarity index (SiD) between E.coli T4 virus and E.coli

#First Calculate the Relative Synonymous Codon Usage (RSCU) for both of them
rscu.T4 <− RSCU.values(fasta.T4)
rscu.Ecoli <− RSCU.values(fasta.Ecoli)

# Then, the SiD could be calculated as
SiD <− SiD.value(rscu.Ecoli, rscu.T4)


SiD measures the effect of the codon usage bias of the *E. coli* on *E. coli* T4 virus. In general, SiD ranged from 0 to 1 with higher values indicating that the host has a dominant effect on the usage of codons. In this example, SiD is approximately equal to 0.491. Which means that E *. coli* does not dominate *E. coli* T4 CUB. Also, this code generates RSCU values for each codon in each gene from both organisms and can be used for further analysis.

## Conclusions

vhcub depends only on DNA sequences as input and can compute different measures of CUB for viruses, such as ENc, CAI, SCUO, and RCDI ( Table 1). It can also be used to study the association between viruses and their hosts’ RSCU and SiD. There are many possible directions for future work; further versions will execute more indices, plots, and statistical analysis, to facilitate the workflow for examining the adaptations of viruses’ CUB in the R environment.

## Data availability

*Escherichia* virus T4 fasta file: ftp://ftp.ncbi.nlm.nih.gov/genomes/all/GCF/000/836/945/GCF_000836945.1_ViralProj14044

*Escherichia coli* fasta file: ftp://ftp.ncbi.nlm.nih.gov/genomes/all/GCF/000/005/845/GCF_000005845.2_ASM584v2/GCF_000005845.2_ASM584v2_cds_from_genomic.fna.gz

## Software availability

Software available from: https://CRAN.R-project.org/package=vhcub

Source code available from: https://github.com/AliYoussef96/vhcub

Archived source code as at time of publication: http://doi.org/10.5281/zenodo.3572391 ^18^

License: GPL-3
