# Supplementary material for: vhcub: Virus-host codon usage co-adaptation analysis
Source: F1000Res. 2019 Dec 23;8:2137. [Version 1] doi: 10.12688/f1000research.21763.1 (PMC7104870; doi:10.12688/f1000research.21763.1)
Supplement: Peer review history

# Peer review: Reviewer response for version 1

**DOI:** 10.5256/f1000research.23991.r61560

**Published:** 2020-03-27

**Authors:** Adriana Patricia Corredor-Figueroa, Oscar Leonardo Ramírez Suárez

**Affiliations:** 1 Ginic-Hus Group Universidad ECCI, Bogotá, Colombia; 2 Universidad ECCI, Bogota, Colombia

- From the technical point of view, the vhcub R package looks quite reliable and well supported. However, there is only one example illustrating it. Moreover, this example shows the mean value for SiD in its range (i.e., 0.491 or approx. 0.5), which is great but makes us wondering if this package could give expected values for other cases. If that is possible, could the authors include a couple of examples where the SiD value were below 0.5 and above 0.5?
- The tool is very interesting and captivating. The advantages that R offers are infinite, so I consider that it would be invaluable to exploit the output that R offers. It is clear in the article that the algorithm only allows the entry of DNA sequences in Fasta format, although there are other very simple tools to use to transcribe from RNA to DNA, or from RNA- to RNA + and DNA, it would be very nice to use the same R package to carry out this step, especially considering that from biological tests we not only analyze one sequence but many. To the same extent, I consider that the figures presented in the article should be more discussed from a biological point of view, they could be more informative.

We confirm that we have read this submission and believe that we have an appropriate level of expertise to confirm that it is of an acceptable scientific standard.

# Peer review: Reviewer response for version 1

**DOI:** 10.5256/f1000research.23991.r58975

**Published:** 2020-02-11

**Authors:** Raj Kumar Singh

**Affiliations:** 1 Facility for Research and Training on Bioassays and Biosensor, Division of Veterinary Biotechnology, ICAR-Indian Veterinary Research Institute [Deemed University], Izatnagar, Uttar Pradesh, India

Viruses in the course of their evolution would optimize their codon usage to their hosts. They rely on the tRNA pool of their hosts in the translation process. Though tools for analyzing the codon usage of organisms are available, none of them focus on examining the codon usage co-adaptation between viruses and their hosts. This software, *vhcub, *is a tool used to analyze the co-adaptation of codon usage between a virus and its host. This may also help to predict the possible mutations that would accumulate in the virus vis - a - vis its host(s), thereby showing the readiness for the control and prevention of the disease.

 General comments

 1. Corrections in the text

-  Spelling of formate may be corrected to format in the second column X first row of Table 1
- Please define df.fasta
- In Third column X sixth row and Third column X eight row are one and the same - please explain or correct

Specific

 Whether it can be used in Eukayotes?

I confirm that I have read this submission and believe that I have an appropriate level of expertise to confirm that it is of an acceptable scientific standard.

# Response

**Published:** 2020-02-11

**Authors:** Ali Mostafa

**Affiliations:** Cairo University, Egypt

**General comments (Corrections in the text)**

- Comment: The spelling of formate may be corrected to format in the second column X first row of Table 1.      

           Response: I will make this correction during the article revisions.

- Comment: Please define df.fasta

           Response: (df.host) as well as (df.virus) are just variables names for data frames holds host genes and virus genes, respectively. The definition will be added during the article revisions.

- Comment: In Third column X sixth row and Third column X eight row are one and the same - please explain or correct

           Response: In the third column X eight row. It will be corrected from ENc to RCDI.

**Specific comments **

- Comment: Whether it can be used in Eukaryotes?

            Response: The translation codon table (The Genetic Codes Tables) number could be changed to any table number (As defined by NCBI https://www.ncbi.nlm.nih.gov/Taxonomy/Utils/wprintgc.cgi), in vhcub. For example in the function named ( CAI.values() ), one can pass an argument genetic.code="11" for bacterial codon table or genetic.code="1" for eukaryotes. Hence, yes, the host can be Prockayotic or Eukaryotic (vhcub can be used for Eukaryotes).

# Response

**Published:** 2020-02-13

**Authors:** Raj Kumar Singh

**Affiliations:** Indian Veterinary Research Institute, India

The authors have accepted to do the necessary changes in the revised version and as well they have answered to my query.
